# Editorial: Modeling Human Potential Across the Lifespan

**DOI:** 10.3389/fpsyg.2020.00106

**Published:** 2020-02-04

**Authors:** Michael J. Stones, Joe Baker

**Affiliations:** ^1^Department of Psychology, Lakehead University, Thunder Bay, ON, Canada; ^2^School of Kinesiology and Health Science, York University, Toronto, ON, Canada

**Keywords:** sport, expertise, creativity, successful aging, age trends

Ebbinghaus (1908) wrote that although “psychology has a long past, yet it's real history is short” (p. 3). The same applies to a scientific understanding of human potential. Simonton (2001) noted a debt owed to nineteenth century polymaths Adolphe Quételet and Francis Galton.

Quetelet (1968) was the first to report quantitative measures of creativity and to discuss bell-shaped distributions of human characteristics. Galton (1869), recognized the significance of the right-side extreme of the normal curve. His research on familial relationships among such high functioning individuals brought the nature vs. nurture controversy into the realm of scientific study.

Models that influenced scientific understandings of lifespan human potential during the past half-century include the sustained deliberate practice model (Ericsson et al., 1993), the successful aging model (Rowe and Kahn, 1987), Simonton's (1990) model of creativity, and statistical models related to adulthood and aging (Schaie and Hertzog, 1985). Articles in this Research Topic aim to advance such modeling primarily with respect to sport.

## The Sustained Deliberate Practice Model of Expertise

This model suggests that the primary requirement for the development of expertise is sustained deliberate practice over a prolonged time period. Studies reported here discuss advances in such modeling.

Kennedy and Fairbrother examined the relevance of this model to the infrequently studied field of disability sport. A sample of accomplished quad rugby players provided retrospective accounts of time spent in deliberate practice, competitive play, and daily life activities for 2-year intervals over their careers. The findings supported the model, with times spent practicing similar to that reported for other types of expertise. An unexpected finding was affective neutrality when engaging in deliberate practice. The reason for this finding is unclear.

Baker et al. proposed a distinction between *expert* and *eminent* athletes. They discuss four indicators of eminence able identify elite minorities in various sports. These indicators also show convergent validity. Their discussion focuses on the following issues: limitations of small sample sizes for eminent athletes; whether the latter are outliers or possess special characteristics; practical implications for programming meant to identify winners rather than able competitors.

McCue et al. studied life history and practice history profiles that previously supported the sustained deliberate practice model. They report 17 distinct pathways toward entry into the Major League Baseball (MLB). They divided these pathways into three categories depending on the institution that immediately preceded MLB selection. Given the diversity among pathways, the authors suggest MLB selection might profitably take account of data from high school and post-secondary institutions to supplement draft selection data.

Buszard et al. reviewed issues related to the relatively neglected topic of scaling in junior sport. For example, the use of playing fields and equipment designed for adults may be reasons for high drop-out and slower development of expertise during pre-adolescence. The authors propose that age-appropriate scaling in junior sport can provide administrators, scientists, and researchers with opportunities to enhance enjoyment and foster expertise regardless of initial ability level.

## The Successful Aging Model

This model distinguished between effects due to aging (*Successful Aging*), disuse (*Usual Aging*), and disease (*Secondary Aging*). Subsequent adaptations focused on engagement and attitudes as prerequisites for successful aging.

Carr and Weir used a mixed-methods design to evaluate “how” and “why” engagement profiles change in later life. The quantitative “how” findings included a decrease in productive and active leisure pursuits, with stability in social and passive leisure activities. The qualitative “why” findings provide five categories of reasons for change: health and physiological limitations; death of someone close or important; perceived change in personal freedom; altered level or direction of desires; and external changes.

Although sport has positive implications for successful aging, Littlejohn and Young point out that “Little is known about whether messaging interventions can motivate sport activity” (p. 1). They compared effects of different types of messaging on beliefs about outcomes associated with sports participation. The messages to different groups of middle-aged participants were about (1) the benefits of sport or (2) the preceding plus information about barriers to participation. A control group received no such messaging. They found no differences in outcomes between the messaging groups, although their combination showed some difference from the control group. The authors discuss possible reasons for the low effects of messaging.

Horton et al. interviewed experienced, male Master athletes, experienced to determine attitudes about “sport as a social comparison” and “downward comparison” with non-athletes. Findings for the former indicated that competitive provided them with enhanced motivation to train and prepare. Findings for the latter fell into two categories of “resisting loss” and “assigning blame” (i.e., they believed that poor health related to poor lifestyle rather than extrinsic factors). The authors conclude that social comparison had positive implications for personal well-being but negatively stereotyped non-athletic people.

## Models of Creativity

Traditional psychological perspectives on creativity in sport were disparaging about its significance. Simonton (2001) wrote that: “… it is rare for creativity to carry the primary weight in attaining eminence as an athlete or coach in sports” (p. 183). Ericsson et al. (2007) considered that: “Most real-world forms of creative achievement have not yet been successfully measured by objective methods (p.39). However, Vaughan et al. confront such perspectives in this Research Topic. Their article proposed that “creative moments, skill and more generally talent in sport, are not traits possessed by individuals alone but … properties of the athlete-environment system shaped by changing constraints.” They advocate a trans-disciplinary perspective that removes disciplinary boundaries” and thereby provides holistic models. A proviso is reciprocity in communicative influence among academics, practitioners and athletes. This paradigm brings forth new challenges and opportunities, particularly with regard to practical applications.

## Statistical Models of Age Trends in Masters Sport

Nikolaidis et al. break new ground in modeling the relationship between age and performance in their study of two distances in the Duathlon World Championships. They analyzed the percentage of race time spent in cycling against with gender, age, and performance quartile groups The findings indicate (1) slower race times with a greater proportion expended on cycling by women than men; (2) proportionately more time for cycling by younger and faster quartile groups; (3) interactions between age and performance quartile groups only in men at the shorter distance. The findings are consistent with other research that showed low age loss in cycling compared to running.

Stones analyzed archival data on the world's best 100 performance times within age group by gender categories for Masters marathon running. One purpose was to compare goodness of fit between traditional forms of analysis with mixed modeling that took account of repeated measures (i.e., age changes), date of performance (i.e., historical trend), and different cohort expressions (i.e., “birth cohort” or “entry cohort,” where the latter represents age at entry into the database). The findings indicated that main effect terms in the model included gender, age changes, and entry cohort differences. The second purpose was to determine whether age deterioration differed with performance level. With performances within each age group by gender category divided into quartiles, the findings indicated greater age losses at slower performance levels for both age changes and entry cohort differences.

The articles included in this Research Topic highlight the diversity of topics currently being explored in this field and the value of this research for extending our understanding of human potential across the lifespan.

## Author Contributions

All authors listed have made a substantial, direct and intellectual contribution to the work, and approved it for publication.

### Conflict of Interest

The authors declare that the research was conducted in the absence of any commercial or financial relationships that could be construed as a potential conflict of interest.
